# Contrasting offspring dependence periods and diving development rates in two closely related marine mammal species

**DOI:** 10.1098/rsos.230666

**Published:** 2024-01-03

**Authors:** Joffrey Jouma'a, Florian Orgeret, Baptiste Picard, Patrick W. Robinson, Henri Weimerskirch, Christophe Guinet, Daniel P. Costa, Roxanne S. Beltran

**Affiliations:** ^1^ Ecology and Evolutionary Biology, University of California Santa Cruz, CA, USA; ^2^ Institute of Marine Sciences, University of California Santa Cruz, CA, USA; ^3^ Marine Apex Predator Research Unit, Department of Zoology, Institute for Coastal and Marine Research, Nelson Mandela University, Gqeberha 6031, South Africa; ^4^ Centre d'Etudes Biologiques de Chizé, UMR 7372 La Rochelle University-CNRS, La Rochelle, France

**Keywords:** diving development, buoyancy, physiological development, *Mirounga* spp., pinniped, comparative analysis

## Abstract

Understanding the ontogeny of diving behaviour in marine megafauna is crucial owing to its influence on foraging success, energy budgets, and mortality. We compared the ontogeny of diving behaviour in two closely related species—northern elephant seals (*Mirounga angustirostris, n* = 4) and southern elephant seals (*Mirounga leonina, n* = 9)—to shed light on the ecological processes underlying migration. Although both species have similar sizes and behaviours as adults, we discovered that juvenile northern elephant seals have superior diving development, reaching 260 m diving depth in just 30 days, while southern elephant seals require 160 days. Similarly, northern elephant seals achieve dive durations of approximately 11 min on their first day of migration, while southern elephant seals take 125 days. The faster physiological maturation of northern elephant seals could be related to longer offspring dependency and post-weaning fast durations, allowing them to develop their endogenous oxygen stores. Comparison across both species suggests that weaned seal pups face a trade-off between leaving early with higher energy stores but poorer physiological abilities or leaving later with improved physiology but reduced fat stores. This trade-off might be influenced by their evolutionary history, which shapes their migration behaviours in changing environments over time.

## 1. Introduction

Foraging behaviour is critical to the survival of juvenile animals [1–3] and has a direct impact on their migration strategies [4–6]. In addition to the distribution of food resources across habitats [7,8], the development of foraging skills is known to influence the timing, duration and routes taken by migrating animals [9,10]. Therefore, understanding how foraging behaviour develops at an early age can provide valuable information about the factors that drive the evolution of migratory behaviours. This early period is particularly crucial, as it is often characterized by a high mortality rate in long-lived species [11,12]. The reduced proficiency [13] aspects of individuals' foraging behaviours [14,15], and predator avoidance abilities [16,17] provide reasonable explanations for early mortality. To survive, individuals must balance the conflicting demands of reducing their vulnerabilities to predation and starvation by efficiently allocating time and energy to predator avoidance and prey capture [18,19]. This is particularly true for air-breathing marine predators that are strongly constrained by the need to return to the ocean surface to replenish oxygen stores [20]. Thus, understanding the development of foraging behaviour and the constraints faced by air-breathing marine predators during the phase of high juvenile mortality can shed light on the complex factors driving the evolution of their diving and migratory behaviours [21,22].

Marine mammals have developed physiological adaptations for oxygen storage and utilization to satisfy the conflicting needs to breathe at the ocean surface but feed at depth [21]. These adaptations allow for slower utilization of endogenous oxygen stores, enabling prolonged diving durations to greater depths. Owing to their smaller size, juveniles are constrained by their reduced oxygen storage [23,24] and cardiovascular systems compared to adults [25,26]. While factors such as learning [27] and environmental conditions [28,29] influence the ontogeny of diving behaviour, physiological limitations are the most constraining [30–33]. However, measuring physiological parameters to study oxygen utilization is challenging in the marine environment, especially in young marine mammals that are known to have low survival rates [15,34].

In addition, marine mammals often migrate over long distances and their migrations are a critical life-history trait [35]. Defined as regular, repeated, and large-scale movements, migrations connect different components of an animal's life cycle [36], such as feeding, breeding and molting sites. Migration is often driven by the need for resources such as food, conspecifics, or habitat that vary in abundance or quality across space and time, requiring some form of energy storage capabilities [37]. Marine mammals have several physiological adaptations for energy allocation and fasting, as well as for the storage and mobilization of fats from blubber stores. As a result, their buoyancy can change significantly during migration. In newly weaned elephant seals (*Mirounga* spp.), changes in body composition can be estimated based on the rate of vertical change in depth, during specific dives where seals spend much of their time passively drifting [38]. Previous research on these so-called ‘drift dives’ revealed that animals undergo substantial changes in body composition during their very first oceanic migration, reducing their relative lipid content by between 7% and 20%, as reflected by changes in their drift rate, crossing neutral buoyancy twice during their entire journey [38].

In migrating species where longitudinal measurements of physiological abilities are not possible, behavioural variation can be used as a proxy for physiological constraints. For example, owing to high mass-specific energy demands [39] and limited experience, juveniles often exhibit behavioural strategies that differ from those of adults [40,41]. Differences between juvenile and adult diving behaviour have been demonstrated in juvenile European shags (*Phalacrocorax aristotelis*) that compensate for their poor foraging abilities by dramatically increasing their foraging time [42]. Another common adaptation in air-breathing marine predators is an increase in dive frequency [43,44]. To compensate for their lower diving abilities, weaned Weddell seal pups (*Leptonychotes weddellii*) have a dive frequency twice that of adults, and spend longer durations at foraging depths to feed successfully, despite diving closer to their anaerobic threshold [45]. Juveniles might therefore push their physiological limits to reduce the chances of both predation and starvation [42,46].

Elephant seals provide a unique opportunity to study the characteristics of the dive response in juveniles owing to their long foraging migrations at sea, allowing remote monitoring of their physiological development throughout several months, as well as a strong natal site fidelity to their terrestrial colonies that facilitates biologger recovery. There are two hemispherically distinct species of elephant seals: the northern elephant seal (*Mirounga angustirostris*), distributed along the west coast of North America [47], and the southern elephant seal (*Mirounga leonina*) which is distributed across the Southern Ocean and on subantarctic islands [48]. Their behaviours on land and at sea are very similar to adults [49], although they forage in distinct habitats. The body sizes and masses of females are similar, but males are larger and heavier in the southern species [49]. Pups differ in both weaning mass and duration, with the northern species weaning at an older age and gaining more mass from maternal care than the southern species [49,50].

Understanding how physiological constraints and behavioural strategies differ across closely related species can provide valuable insights about ecological processes that determine the ontogeny of migration and diving behaviour [22,51]. By comparing the development of diving capacities across the northern and southern elephant seals, we aimed to identify some of the drivers of the ontogeny of diving behaviour [14,52]. Given the differences in weaning mass, duration, and maternal care between northern and southern elephant seals, we predict differences in the ontogeny of diving behaviour between the two species. We anticipate that these differences will manifest in their respective developmental trajectories of diving capacities, drift rate (buoyancy, a proxy of body condition, [53]), providing insights into the unique physiological constraints and behavioural strategies each species employs during early development.

## 2. Material and methods

### 2.1. Study system and animal handling

**Figure 1.**
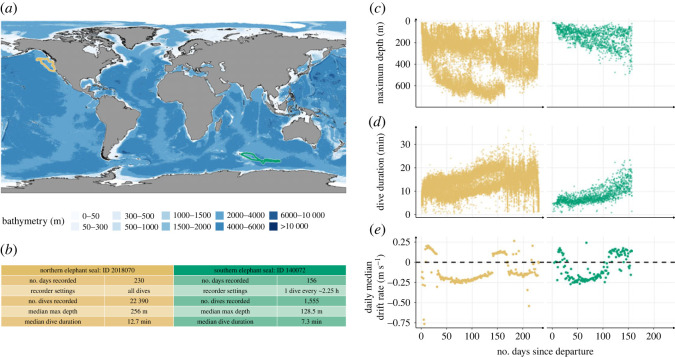
Illustrative data from one representative weanling northern elephant seal (ID: 2018070) and one southern elephant seal (ID: 130072) for comparison. The panels show for each of these two representative seals: (*a*) the migration routes during their first trip to sea from Año Nuevo, California, United States of America, and Kerguelen Island, France, respectively in gold and green; (*b*) a summary of tag set-up and dive characteristics; the development of (*c*) maximum diving depth, (*d*) dive duration, and (*e*) daily median drift rate. Data show clear improvement of diving metrics for both species, with northern elephant seals exhibiting accelerated development in both diving duration and depth.

In 2018, 24 juvenile northern elephant seals (15 females and nine males) from the population at Año Nuevo Reserve, CA, USA (figure 1*a*; 37°5′ N, 122°16′ W) were equipped with an archival time-depth recorder (MK9, Wildlife Computers, measures time, depth, light) to record their very first trip to sea. The tags of only four individuals were recovered when they returned to land. Animals were sedated with an initial injection of tiletamine hydrochloride and zolazepam hydrochloride (Telazol), administered intramuscularly. Immobilization was maintained with intravenous injections of ketamine when needed. Using quick-setting epoxy (Loctite, Epoxy General Purpose), the MK9 tag was affixed to the fur on the centre of the back.

In 2014, 20 juvenile southern elephant seals (10 females and 10 males) from Kerguelen Islands, sub-Antarctic French territories (figure 1*a*; 49°20′ S, 70°20′ E) were equipped with a custom-designed Argos relay satellite tag (SPLASH10-F-2961-DSA tag, Wildlife Computers, hereafter DSA tag) and a smart position transmitting tag (SPOT 293A, Wildlife Computers) that measured diving depth to record their very first trip to sea. The satellite tags of nine individuals were recovered when they returned to land (see [15] for details). Animals were captured with a canvas head-bag and anesthetized using a 1 : 1 combination of tiletamine hydrochloride and zolazepam hydrochloride (Zoletil 100) injected intravenously. Using quick-setting epoxy (Araldite AW 2101), the DSA tag was attached to the fur on top of the head and the SPOT tag to the centre of the back.

### 2.2. Data collection

The MK9 tags attached to northern elephant seals sampled pressure every 4 s during the entire trip to sea (figure 1*b*). One complete dive (max depth >15 m and duration >60 s) was recorded every approximately 2.2 h (figure 1*b*) by the DSA tags, with a pressure sampling rate of 1 Hz.

### 2.3. Data preprocessing

Time-depth records of southern elephant seals were downsampled to 4 s to match the sampling frequency of the northern elephant seals and facilitate comparison. Dive identification and zero offset correction of depth were performed using the IKNOS Toolbox detailed by Robinson *et al*. [54] with Matlab 9.1 (The MathWorks, Natick, MA, USA). Dives were identified as excursions from the surface reaching a maximum depth of at least 10 m and lasting a minimum duration of 30 s.

For northern elephant seals, location data were obtained from the light-level data using the Wildlife Computers GPE3 algorithm, with the exception of seal ID 2018074 in which the light levels were corrupted. Using the aniMotum R package [55], we fitted a correlated random walk with a maximum travel rate of 3 m s^−1^ on our location data to re-estimate the animal's position every hour in order to get a regular time step and handle gaps. The time of day was calculated using the function sunriset() from the R package maptools, which estimates night-time and day-time based on date-time and location from the National Oceanic and Atmospheric Administration solar calculator [56].

### 2.4. Dive type classification

Active bottom dives were identified using a method initially developed on northern elephant seals [57], based on an index that identifies and scores putative foraging dives. Because elephant seals exhibit a variety of diving behaviours during the bottom phase of putative foraging dives, most parameters inferred from time-depth profile, such as foraging depth, size and frequency of vertical excursions (or wiggles, i.e. small vertical inflections probably associated with prey pursuit) can vary substantially. The *intensity index* was designed to assess active bottom dives by encapsulating several key traits associated with activity, scaled to the size of the bottom phase. Dives that exhibit many wiggles during the bottom phase, i.e. intensity index higher than 35, were considered as active bottom dives:

$$\mathrm{intensity}\;\mathrm{index}=\frac{W*V}{R}+\left[\left(\frac{W*V}{R}\right)*\left(\frac{V}{T}\right)\right],$$

where *W* is number of wiggles during the bottom phase; *V* is total vertical distance travelled during the bottom phase; *R* is range of depth values during the bottom phase; and *T* is duration of the bottom phase.

Drift dives were identified using a method developed by Robinson *et al*. [58], based on the first derivative (vertical component of velocity) of the time-depth profile. For each dive, a kernel density estimation of the vertical speed was used to find both the drift rate (position of the peak) and the relative proportion of the dive spent drifting at the dominant drift rate (height of the peak). If the height of the peak exceeded a density of 1, a large proportion of the dive took place at the dominant drift rate and was thus classified as a drift dive. For each drift dive a drift rate was then estimated from the dominant peak found on a drift rate kernel density. We summarized these values by calculating median daily drift rates.

Dives were classified benthic if they had (i) minimal bottom phase vertical excursions, (ii) square corners, and (iii) a bottom phase slope close to zero. To characterize the bottom phase vertical excursions, we also calculated a kernel density of bottom phase vertical speed for each dive. If the peak fell within the range of ±0.08 m s^−1^ (nearly flat bottom) and the height of the peak exceeded a density of 1.5 (consistent vertical speed), it was classified as a benthic dive. Additionally, best-fit lines were drawn for the descent phase and the bottom phase, and the intersection between the two lines was checked against the animal's actual trajectory. If the actual trajectory was less than 15 m from the intersection point (when the switch from descent phase to bottom phase was sharp), it was identified as a benthic dive. Occasionally, benthic dives would also register as drift dives because of their very consistent rate of depth change over a large part of the dive, in which case these dives were identified as benthic. The remaining unclassified dives were categorized as transit dives.

### 2.5. Statistical analyses

To assess temporal changes in maximum depth, dive duration and daily median drift rate (figure 1*c,d,e*), each of these parameters were fitted as the response variable in generalized additive mixed-effects models, with the number of days since departure as the explanatory variable, using the function bam() from the R package mgcv [59]. To avoid overfitting, a cubic regression spline with a maximum of six knots was used as a smooth term [29]. To compare differences between the northern and southern elephant seals, we included species as a covariate and a grouping factor in each model; and to account for individual variability, we also included a random effect by individual on the intercept and the slope [60]. To account for the autocorrelation [29], we included a first-order autoregressive model, by specifying the parameter rho as the first lag from the autocorrelation function estimation derived from the acf() function. To estimate age-related increases in the maximum physiological capabilities of the seals, we calculated the 95th daily percentile of dive depth and duration for each individual (table 1).

**Table 1.** Descriptive statistics and visual representations of the dataset's first offshore foraging trip for each northern and southern elephant seal. (For maximum depth (m; note the inverted *y*-axis) and dive duration (min), the trend represents the changes in the daily 95th percentile through time (solid grey line) associated with a linear regression (black dashes). For drift rate (m s^−1^), the daily median was calculated to represent the evolution over time, with positive values in orange and negative in violet. The measurements of length and mass were made at weaning.)

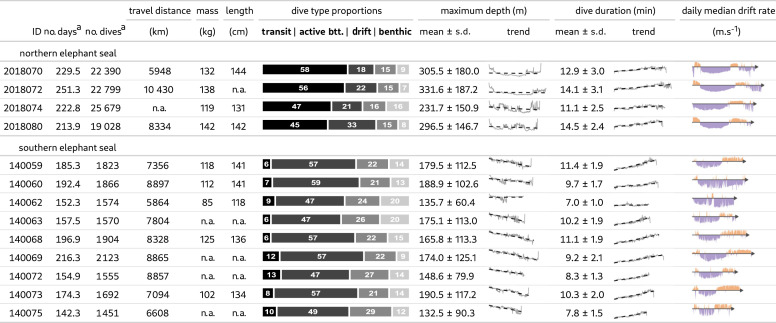

^a^Recorded.

We first calculated these proportions per individual to estimate the proportion of the number of dives for each dive type at the population level. We then averaged them weighted by the total number of dives. Two-sample *z*-tests for proportions without continuity correction were performed to investigate differences in these proportions between day-time and night-time for each species. A two-sided Mann–Whitney *U*-test was used to compare the dive duration and maximum dive depth distribution between these two species. Where appropriate, variables were described using the mean ± standard deviation. All statistical analyses were conducted in R Statistical Software 4.2.2 [61].

## 3. Results

Forty-four weaned juvenile elephant seals from the Año Nuevo and Kerguelen Islands populations were equipped with a time-depth recorder. Of these, 13 instruments were recovered, recording a total of 105 454 dive profiles (four female northern elephant seals; mean weaning mass ± s.d. = 133 ± 10 kg; mean straight length from nose to tail tip ± s.d. = 139 ± 7 cm; and seven female and two male southern elephant seals; mean weaning mass ± s.d. = 108 ± 16 kg; mean straight length from nose to tail tip ± s.d. = 134 ± 9 cm; table 1). During their first foraging trip to sea, northern and southern juveniles spent 229.4 ± 16 days and 174.7 ± 24.7 days (max 251 days) at sea and travelled 8237 ± 2242.6 km and 7741.6 ± 1093.4 km (max 10 430 km) respectively (table 1).

### 3.1. Diving behaviours

**Figure 2.**
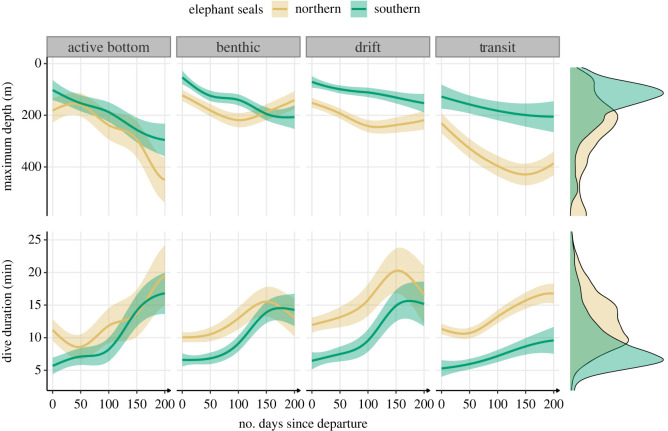
Development of depth and duration across dive types throughout the first trip to sea in northern (*n* = 4) and southern elephant seals (*n* = 9) estimated from generalized additive models. The solid lines represent the means, and the shaded areas represent the 95% confidence intervals. Marginal density plots represent the spread of data across all dive types for each species.

Both species showed similar patterns in the shape of diving development (figure 2). Within a few days of departure, they reached 100 m depth, and dived for more than 5 min (figure 2). Both species reached approximately 20 min by the end of their first trip to sea, the average dive duration of an adult. Juvenile northern elephant seals exhibited significantly superior maximum dive depth (Mann–Whitney test, *U* = 902 643 075, *n*_northern_ = 797 32, *n*_southern_ = 15 387, *p* < 0.0001) and dive duration (Mann–Whitney test, *U* = 897 660 292, *n*_northern_ = 79 732, *n*_southern_ = 15387, *p* < 0.0001) compared to juvenile southern elephant seals. These differences were characterized by longer and deeper dives across all dive types for northern elephant seals throughout their foraging migrations (dive duration: 13.0 ± 4.7 min versus 9.5 ± 4.7 min; maximum dive depth: 289.1 ± 171.8 m versus 167 ± 106.7 m; figure 2).

**Figure 3.**
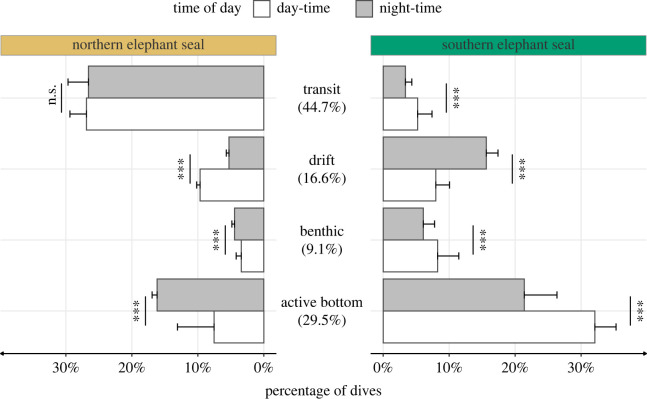
Frequency of dive types across time of day and species. Species-wide statistical tests were based on averages of each individual's dive type proportion weighted by the total number of dives. Percentages in the middle panel represent the frequency of each dive type across species and time of day. Asterisks (***) indicate a significant difference (*p*-value < 0.0001) between day-time and night-time dive proportions within each species (two-sample *z*-test).

For most dive types, a similar temporal increase in maximum dive depth was observed between both species across the first trip to sea (figure 2). Northern pups made transit dives 100–200 m deeper than southern ones (44.7% of all dives; figure 3). For drift dives (9.1% of all dives; figure 3) and benthic dives (16.6% of all dives; figure 3), dive depth and duration were longer for northern elephant seals at the beginning of the trip. Still, southern elephant seals dived deeper and longer after 150 days at sea (figure 2). The depth and duration of active bottom dives (29.5% of all dives; figure 3) were similar between the two species. Active bottom dives reflected more development in depth and duration than other dive types for both species (figure 2). Overall, southern elephant seal diving behaviours were characterized by a lower proportion of transit dives (8.6% versus 53.5%) and a higher proportion of active bottom dives (53.5% versus 23.7%), compared to northern elephant seals (figure 3).

### 3.2. Drift rate

**Figure 4.**
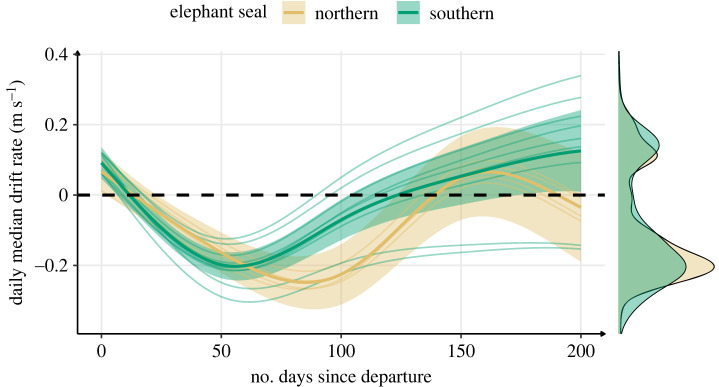
Changes in median drift rate across the first trip to sea in northern (*n* = 4) and southern (*n* = 9) elephant seals estimated from a generalized additive model. The bold solid lines represent the mean species-level responses while the thin lines represent individual-level responses. The shaded areas represent the 95% confidence interval, and the black dashed line indicates neutral buoyancy. Marginal density plots indicate the data spread across the entire migration for each species.

Drift rate reflects seals' buoyancy, and therefore to their body condition [38]. The temporal changes in daily median drift rate were similar across species (figure 4). Both northern and southern elephant seals exhibited positive drift rates immediately after departure. The first phase of the oceanic migration was characterized by a substantial reduction in body condition. Seals reached neutral buoyancy within approximately 10 days, and their body condition continued to decline until day 50 and 75 for southern and northern elephant seals respectively (figure 4). The second phase was characterized by an increase in the daily median drift rate, steeper in southern elephant seals, that slowed down when positive buoyancy was again reached (around day 125 and 137 for respectively southern and northern elephant seals). For northern elephant seals, this step was followed by a third phase where daily median drift rate decreased, crossing neutral buoyancy again around day 175.

### 3.3. Time of day

Day-time and night-time durations were similar across both species, with 13.3 ± 2.4 h and 10.7 ± 2.4 h for northern elephant seals, and 13 ± 3.1 h and 11 ± 3.1 h for southern elephant seals. Except for transit dives in the northern elephant seal (two-sample *z*-test for proportion, *z* = 1.51, *p* = 0.876), the proportion of dive types differed significantly between day and night for both species (figure 3). While southern elephant seals performed more active bottom dives during the day (32.1% versus 21.4%), northern elephant seals exhibited the opposite with 16.2% night-time active bottom dives and 7.5% day-time active bottom dives. The same difference was found for drift dives, where northern elephant seals performed 9.7% of these dives during the day and 5.3% at night, while southern elephant seals conducted 8.0% and 15.6% during day-time and night-time (figure 3).

## 4. Discussion

Despite their similar size and behaviours as adults, we discovered striking differences in development of diving abilities during the first foraging migration between northern and southern elephant seals. While previous studies have separately described the ontogeny of these behaviours in each species [14,15,38,62], this is, to our knowledge, the first time that both species have been studied together using high-frequency recorders, which facilitated standardized analysis of behavioural and physiological development and allowed for meaningful comparisons. Post-weaning elephant seals could reach adults’ average dive depth and duration by the end of their first trip to the sea (i.e. upon their return to shore). Both species showed similar patterns of diving development at sea. However, juvenile northern elephant seals developed much faster overall, which allowed them to undertake deeper and longer dives earlier in the foraging migration. The observed differences in the ontogeny of diving and migration behaviour between the two species suggest that key ecological processes may be at play [10,63], including maternal care trade-offs, physiological constraints, habitat conditions, predation risk, diet preferences and selection pressures associated with limited body conditions Each of these aspects will be discussed in detail in the following sections.

### 4.1. Maternal care

This difference in development of diving abilities could be linked to different durations of maternal dependency periods, which are essential for muscle maturation in pinnipeds [64–66]. With a lactation duration of 24 to 28 days [49], northern elephant seals nurse for a few more days than southern elephant seals (22 to 23 days; [34]) which could allow them to develop larger fat and endogenous oxygen stores. In addition to the lactation period, the post-weaning fast is also vital for the development of diving physiology [24]. For instance, in grey seals (*Halichoerus grypus*), the total body mass-specific oxygen stores increase by 35% and the calculated aerobic dive limit by 23% during this period [24]. Both northern and southern elephant seals also increase their mass-specific blood volume during the post-weaning fast [67,68]. Northern elephant seals have a longer post-weaning fast than southern elephant seals (56 ± 16 versus 37 ± 11 days, [69]), allowing them to build greater oxygen stores. The extended nursing and post-weaning fast probably allow northern elephant seals to mature physiologically, which enables them to dive deeper and longer to some extent. However, this may not be enough to explain the substantial differences in diving abilities between the two species. Further studies are needed to assess comparative studies of blood volume and haematocrit concentration in both species [22].

### 4.2. Physiology

Like other juvenile marine mammals [45], post-weaning elephant seals are limited in diving physiology and thus behaviour, owing to lower mass-specific body oxygen stores, higher mass-specific metabolic rate [68], and smaller body sizes compared to adults. In Australian sea lions (*Neophoca cinerea*), 3-year-old juveniles only reach 78% of adult total oxygen stores [31]. The same was found in juvenile Steller sea lions (*Eumetopias jubatus*) which have low mass-specific total body oxygen stores until they reach two years of age [70]. Even after this age, their calculated aerobic diving limit was still lower than that of adults, probably owing to their smaller size and higher mass-specific metabolic rates. Because elephant seals reach adult size at 3–4 years of age, post-weaning animals are expected to have lower diving capacities than adults. However, the difference in diving behaviour observed between post-weaning northern and southern elephant seals disappears with age. Adults from both species show similar dive durations (respectively 23.1 ± 2.6 min, [54] and 20.7 ± 4.8 min, [71]) and dive depths (respectively 516.0 ± 53.2 m, [54] and 542.0 ± 226.0 m, [71]). Considering how constrained newly weaned elephant seals are in their anatomy and physiology, it is remarkable that, under certain conditions, they are capable of reaching the typical diving depth and duration of adults, which exceed those of most pinnipeds [72,73]. Although adult southern elephant seals appear to achieve greater maximum dive depths and durations compared to adult northern elephant seals [74], the development of these differences was not apparent during the first trip to sea.

### 4.3. Habitat

On land, maternal dependency and post-weaning fast periods are both affected by the environment surrounding the breeding site including weather conditions, female density, and distance to male fights. These influences might work differently for northern and southern elephant seals given the observed differences in lactation duration. At sea, adults of both species target mesopelagic prey within oceanic structures such as cyclonic and anticyclonic eddies [75,76], but little is known about the foraging habitat of juveniles. Both species forage in different environments: the northeast Pacific Ocean and the Southern Ocean. Northern elephant seals feed in the northeast Pacific Ocean, a region mainly characterized by two eddy hotspots with the Gulf of Alaska and the California Current, along with the North Pacific Transition Zone where the North Pacific subtropical and subpolar gyres meet [54]. By contrast, southern elephant seals feed in the Southern Ocean, an annular ocean characterized by the strong Antarctic Circumpolar Current (ACC) accompanied by the Subantarctic, the Polar, and the Southern ACC front. The oceanographic characteristics of these two oceans play an important role in shaping their respective ecosystems and influence distributions, abundances and behaviours of prey [77], and it is likely that contrasting diving abilities might have evolved in response to these varying conditions.

### 4.4. Predation

Better diving abilities are thought to provide an advantage for avoiding predators and/or accessing prey. Great white sharks (*Carcharodon carcharias*) and killer whales (*Orcinus orca*) are the main predators of juvenile northern elephant seals [78,79]; southern elephant seals are predated by killer whales [80–82] as well as sleeper sharks (*Somniosus antarcticus*) [83]. The resulting landscape of fear could therefore differ between species owing to variations in the habitat and distribution of their predators. For example, killer whales and great white sharks are known to forage close to shore or in continental shelf waters [84], whereas sleeper sharks are generally found in deeper waters [75]. As a result, both species face high predation pressure inshore and near colonies where seal density is high, but southern elephant seals may face larger predator exposure, as the Kerguelen plateau extends the continental shelf much further than that of Año Nuevo. This difference in predation pressure may have shaped the ontogeny of diving behaviour differently in these species as they adapt to their respective environments and predators.

### 4.5. Diet

From a foraging perspective, physiological diving constraints can also represent a disadvantage for young animals feeding on the same prey with the same three-dimensional distribution as adults [70]. This is the case in elephant seals, where adults feed mainly at the bottom of their dives, at depths beyond the reach of juveniles, especially at the beginning of their migration [85]. If the physiological constraints are too great, then juveniles may feed on different prey to meet their energetical needs [45]. Unlike the adults that mostly feed on fish and squid [86], nitrogen stable isotope signatures reveal that first year juvenile southern elephant seals feed on lower trophic levels [87,88], probably with a higher proportion of crustaceans in their diet [89,90]. In northern elephant seals, adults feed mainly on fishes and to a lesser extent on squid [91,92], but information on the diet of juvenile seals is not available. Juveniles must learn to find, catch and handle prey while managing a limited diving capacity [14]. When weaned, the large fat reserves act as a buffer while they learn to forage on their own. The substantial decrease in their body condition suggests that it takes a few weeks before the pups ingest more calories than they expend trying to find food. Given their poor diving abilities and high energy requirements, the higher proportion of crustaceans may represent the most feasible option while they learn to catch and handle fish and squid.

### 4.6. Body condition

Diet and foraging success influence buoyancy through changes in body composition, making it paramount to study the foraging ecology of diving animals. Variations in buoyancy directly impact both elephant seal species' swimming effort (or stroke-per-meter), with a minimum foraging effort occurring when seals achieve neutral buoyancy [93–95]. Changes in buoyancy also influence parameters such as the duration of their haul-outs and selection of foraging habitat, as well as seals’ decisions to continue foraging or return to the land [14]. In our study, all northern elephant seals initiated their return to land after switching from negatively to positively buoyant, which is consistent with observations of southern elephant seals [14]. This decision to return may also reflect the sufficient development of lean muscular tissue, after which excess energy is stored as fat [14]. There are strong implications for storing optimal amounts of fat to cope with food shortages when deviating from neutral buoyancy, particularly in capital breeding species in which fat storage during reproduction provides clear fitness benefits [96].

Unlike adults, newly weaned elephant seals were positively buoyant at the beginning of their first trip to sea. Therefore, by delaying their timing of departure, weaned pups may face a trade-off between an early departure with greater energy stores but poorer physiological capabilities and a late departure that would extend their post-weaning fast, with improved diving physiology but reduced fat stores [97]. In southern elephant seals, heavier juveniles that nursed longer have better diving capabilities, suggesting that smaller individuals are more constrained by their physiological capacities [98]. This leads to the assumption that having a better body condition at departure should facilitate increased foraging ability or reduced vulnerability to predation, all conferring a survival advantage [98]. However, while accounting for the small sample size, no relationship was found between survival outcome and body condition in post-weaned southern elephant seals during their first trip to sea [15], suggesting that other factors such as predation are likely to contribute to pup mortality during their first year at sea. Thus, prey and predator distribution heterogeneity may overwhelm the benefits of fat stores in elephant seals.

### 4.7. Limitations

Despite knowledge of potential drivers of the ontogeny of diving behaviour, such as maternal care, physiological processes, predation, habitat and diet, it remains difficult to elucidate why juvenile southern elephant seals perform significantly more active bottom dives and fewer transit dives than their congenerics (figure 3). With the same dive type identification method used in both species, the observed difference suggests that the two species use different feeding strategies. Because the dive type classification algorithm was developed for northern elephant seals, the disparity in the observed proportions of dive types could underestimate time spent foraging by southern elephant seals. Alternatively, assuming their energy intake must be similar, the lower proportion of active bottom dives in northern elephant seals may indicate they are feeding on more abundant or energy-rich prey. While active bottom dives have been shown to correlate well with whole-trip foraging success [58,99], they do not, by definition, account for foraging dives where no vertical excursions (wiggles) are performed, such as those along the seafloor. Further comparative studies of diet and fine-scale movement, such as using accelerometers to infer feeding behaviour, are needed to identify the reasons behind this difference in proportional dive types.

## 5. Conclusion

The study of juveniles is crucial because the selection pressures they experience are fundamental for understanding population dynamics. Understanding the mechanisms behind the high mortality typically observed at age 0–1 (approx. 33%–43% for northern elephant seals, [100]; approximately 45% for southern elephant seals, [15]) is essential to understand population age-structures and growth rates [101]. During their first trip to sea, elephant seals must transition from maternal care on land to learning how to navigate, forage, and survive on their own, in a three-dimensional and highly heterogeneous environment by improving their physiological capacities, foraging efficiency and predator avoidance strategies. Our study highlights the importance of comparing the ontogeny of related species to elucidate significant ecological processes in life-history strategies. By comparing the two elephant seal species and analysing their behaviour at sea using bio-logging data, we identified potential drivers of the ontogeny of their diving behaviour. Our results suggest that, although a variety of factors may play a role, periods of lactation and fasting appear to be the primary drivers. To obtain a broader picture, future studies should explore the physiological maturation that occurs during the post-weaning fast, investigate the diet of post-weaning pups and examine changes in their environmental conditions.

## Data Availability

All diving data are available in a Dryad public data repository [102] and the code in a Zenodo repository [103]. Electronic supplementary material is available online [104].

## References

[1] Danchin E, Giraldeau L-A, Cézilly F. (eds) 2008 Behavioural ecology: an evolutionary perspective on behaviour. Oxford, UK: Oxford University Press.

[2] MacArthur RH, Pianka ER. 1966 On optimal use of a patchy environment. Am. Nat. **100**, 603-609.

[3] Emlen JM. 1966 The role of time and energy in food preference. Am. Nat. **100**, 611-617. (10.1086/282455)

[4] Hassell MP, Southwood TRE. 1978 Foraging strategies of insects. Annu. Rev. Ecol. Syst. **9**, 75-98.

[5] Beauchamp G. 2006 Spatial, temporal and weather factors influencing the foraging behavior of migrating semipalmated sandpipers. Waterbirds Int. J. Waterbird Biol. **29**, 221-225.

[6] Rueda-Uribe C, Lötberg U, Åkesson S. 2022 Foraging on the wing for fish while migrating over changing landscapes: traveling behaviors vary with available aquatic habitat for Caspian terns. Mov. Ecol. **10**, 9. (10.1186/s40462-022-00307-8) 35236399 PMC8892754

[7] Boscarino BT, Rudstam LG, Mata S, Gal G, Johannsson OE, Mills EL. 2007 The effects of temperature and predator—prey interactions on the migration behavior and vertical distribution of *Mysis relicta*. Limnol. Oceanogr. **52**, 1599-1613. (10.4319/lo.2007.52.4.1599)

[8] Polovina JJ, Howell E, Kobayashi DR, Seki MP. 2001 The transition zone chlorophyll front, a dynamic global feature defining migration and forage habitat for marine resources. Prog. Oceanogr. **49**, 469-483. (10.1016/S0079-6611(01)00036-2)

[9] Collet J, Prudor A, Corbeau A, Mendez L, Weimerskirch H. 2020 First explorations: ontogeny of central place foraging directions in two tropical seabirds. Behav. Ecol. **31**, 815-825. (10.1093/beheco/araa028)

[10] Stewart BS. 1997 Ontogeny of differential migration and sexual segregation in northern elephant seals. J. Mammal. **78**, 1101-1116. (10.2307/1383053)

[11] Gaillard J-M, Festa-Bianchet M, Yoccoz NG. 1998 Population dynamics of large herbivores: variable recruitment with constant adult survival. Trends Ecol. Evol. **13**, 58-63. (10.1016/S0169-5347(97)01237-8) 21238201

[12] Sæther B-E et al. 2013 How life history influences population dynamics in fluctuating environments. Am. Nat. **182**, 743-759. (10.1086/673497) 24231536

[13] Wunderle JM. 1991 Age-specific foraging proficiency in birds. Curr. Ornithol. **8**, 273-324.

[14] Orgeret F, Cox SL, Weimerskirch H, Guinet C. 2019 Body condition influences ontogeny of foraging behavior in juvenile southern elephant seals. Ecol. Evol. **9**, 223-236. (10.1002/ece3.4717) 30680109 PMC6341977

[15] Cox SL, Authier M, Orgeret F, Weimerskirch H, Guinet C. 2020 High mortality rates in a juvenile free-ranging marine predator and links to dive and forage ability. Ecol. Evol. **10**, 410-430. (10.1002/ece3.5905) 31988734 PMC6972805

[16] Cooke F, Findlay CS, Rockwell RF. 1984 Recruitment and the timing of reproduction in lesser snow geese (*Chen caerulescens caerulescens*). The Auk **101**, 451-458. (10.1093/auk/101.3.451)

[17] Majluf P. 1992 Timing of births and juvenile mortality in the South American fur seal in Peru. J. Zool. **227**, 367-383. (10.1111/j.1469-7998.1992.tb04400.x)

[18] Weathers WW, Sullivan KA. 1989 Juvenile foraging proficiency, parental effort, and avian reproductive success. Ecol. Monogr. **59**, 223-246. (10.2307/1942600)

[19] Brown JS. 1999 Vigilance, patch use and habitat selection: foraging under predation risk. Evol. Ecol. Res. **1**, 49-71.

[20] Butler PJ, Jones DR. 1997 Physiology of diving of birds and mammals. Physiol. Rev. **77**, 837-899. 9234967 10.1152/physrev.1997.77.3.837

[21] Ponganis PJ. 2015 Diving physiology of marine mammals and seabirds. Cambridge, UK: Cambridge University Press. (10.1017/CBO9781139045490)

[22] Weitzner EL, Fanter CE, Hindle AG. 2020 Pinniped ontogeny as a window into the comparative physiology and genomics of hypoxia tolerance. Integr. Comp. Biol. **60**, 1414-1424. (10.1093/icb/icaa083) 32559283

[23] Burns JM, Costa DP, Frost K, Harvey JT. 2005 Development of body oxygen stores in harbor seals: effects of age, mass, and body composition. Physiol. Biochem. Zool. PBZ **78**, 1057-1068. (10.1086/432922) 16228944

[24] Noren SR, Iverson SJ, Boness DJ. 2005 Development of the blood and muscle oxygen stores in gray seals (*Halichoerus grypus*): implications for juvenile diving capacity and the necessity of a terrestrial postweaning fast. Physiol. Biochem. Zool. **78**, 482-490. (10.1086/430228) 15957103

[25] Burns JM, Castellini MA. 1996 Physiological and behavioral determinants of the aerobic dive limit in Weddell seal (*Leptonychotes weddellii*) pups. J. Comp. Physiol. B **166**, 473-483. (10.1007/BF02338290)

[26] Rutishauser MR, Costa DP, Goebel ME, Williams TM. 2004 Ecological implications of body composition and thermal capabilities in young Antarctic fur seals (*Arctocephalus gazella*). Physiol. Biochem. Zool. **77**, 669-681. (10.1086/421749) 15449238

[27] Grecian WJ, Lane JV, Michelot T, Wade HM, Hamer KC. 2018 Understanding the ontogeny of foraging behaviour: insights from combining marine predator bio-logging with satellite-derived oceanography in hidden Markov models. J. R. Soc. Interface **15**, 20180084. (10.1098/rsif.2018.0084) 29875281 PMC6030624

[28] Lea M-A, Johnson D, Ream R, Sterling J, Melin S, Gelatt T. 2009 Extreme weather events influence dispersal of naive northern fur seals. Biol. Lett. **5**, 252-257. (10.1098/rsbl.2008.0643) 19147444 PMC2665815

[29] Grecian WJ et al. 2022 Environmental drivers of population-level variation in the migratory and diving ontogeny of an Arctic top predator. R. Soc. Open Sci. **9**, 211042. (10.1098/rsos.211042) 35316952 PMC8889203

[30] Boyd IL, Croxall JP. 1996 Dive durations in pinnipeds and seabirds. Can. J. Zool. **74**, 1696-1705. (10.1139/z96-187)

[31] Fowler SL, Costa DP, Arnould JPY, Gales NJ, Burns JM. 2007 Ontogeny of oxygen stores and physiological diving capability in Australian sea lions. Funct. Ecol. **21**, 922-935. (10.1111/j.1365-2435.2007.01295.x)

[32] Kooyman GL. 2011 Diverse divers: physiology and behavior. Berlin, Germany: Springer.

[33] Tift MS, Ranalli EC, Houser DS, Ortiz RM, Crocker DE. 2013 Development enhances hypometabolism in northern elephant seal pups (*Mirounga angustirostris*). Funct. Ecol. **27**, 1155-1165. (10.1111/1365-2435.12111) PMC3811961 24187422

[34] Le Boeuf BJ, Morris P, Reiter J. 1994 Juvenile survivorship of northern elephant seals. In Elephant seals - population ecology, behavior, and physiology (eds BJ Le Boeuf, RM Laws), pp. 121-136. Berkeley, CA: University of California Press. (10.1525/9780520328150-009)

[35] Boyd IL. 2004 Migration of marine mammals. In Biological resources and migration (ed. D Werner), pp. 203-210. Berlin, Germany: Springer. (10.1007/978-3-662-06083-4_20)

[36] Dingle H. 2014 Migration: definition and scope. In Migration: the biology of life on the move (ed. H Dingle), pp. 13-23. Oxford, UK: Oxford University Press. (10.1093/acprof:oso/9780199640386.003.0002)

[37] Milner-Gulland EJ, Fryxell JM, Sinclair ARE. 2011 Animal migration. Oxford, UK: Oxford University Press.

[38] Biuw M, McConnell B, Bradshaw CJA, Burton H, Fedak M. 2003 Blubber and buoyancy: monitoring the body condition of free-ranging seals using simple dive characteristics. J. Exp. Biol. **206**, 3405-3423. (10.1242/jeb.00583) 12939372

[39] Schmidt-Nielsen K. 1997 Animal physiology: adaptation and environment, 5th edn. Cambridge UK: Cambridge University Press.

[40] Fowler SL, Costa DP, Arnould JPY, Gales NJ, Kuhn CE. 2006 Ontogeny of diving behaviour in the Australian sea lion: trials of adolescence in a late bloomer. J. Anim. Ecol. **75**, 358-367. (10.1111/j.1365-2656.2006.01055.x) 16637989

[41] Fowler SL, Costa DP, Arnould JPY. 2007 Ontogeny of movements and foraging ranges in the Australian sea lion. Mar. Mammal Sci. **23**, 598-614. (10.1111/j.1748-7692.2007.00134.x)

[42] Daunt F, Afanasyev V, Adam A, Croxall JP, Wanless S. 2007 From cradle to early grave: juvenile mortality in European shags *Phalacrocorax aristotelis* results from inadequate development of foraging proficiency. Biol. Lett. **3**, 371-374. (10.1098/rsbl.2007.0157) 17504733 PMC2390668

[43] Zeno RL, Crocker DE, Hassrick JL, Allen SG, Costa DP. 2008 Development of foraging behavior in juvenile northern elephant seals. J. Zool. **274**, 180-187. (10.1111/j.1469-7998.2007.00371.x)

[44] Leung ES, Chilvers BL, Nakagawa S, Robertson BC. 2014 Size and experience matter: diving behaviour of juvenile New Zealand sea lions (*Phocarctos hookeri*). Polar Biol. **37**, 15-26. (10.1007/s00300-013-1405-6)

[45] Burns JM. 1999 The development of diving behavior in juvenile Weddell seals: pushing physiological limits in order to survive. Can. J. Zool. **77**, 737-747. (10.1139/z99-022)

[46] Orgeret F, Weimerskirch H, Bost C-A. 2016 Early diving behaviour in juvenile penguins: improvement or selection processes. Biol. Lett. **12**, 20160490. (10.1098/rsbl.2016.0490) 27484650 PMC5014042

[47] Lowry M. 2014 Abundance, distribution, and population growth of the northern elephant seal (*Mirounga angustirostris*) in the United States from 1991 to 2010. Aquat. Mamm. **40**, 20-31. (10.1578/AM.40.1.2014.20)

[48] Hindell MA et al. 2016 Circumpolar habitat use in the southern elephant seal: implications for foraging success and population trajectories. Ecosphere **7**, e01213. (10.1002/ecs2.1213)

[49] Le Boeuf BJ, Laws RM. 1994 Elephant seals: population ecology, behavior, and physiology. Berkeley, CA: University of California Press.

[50] Le Bœuf BJ, Condit R, Reiter J. 1989 Parental investment and the secondary sex ratio in northern elephant seals. Behav. Ecol. Sociobiol. **25**, 109-117. (10.1007/BF00302927)

[51] Piotrowski ER, Tift MS, Crocker DE, Pearson AB, Vázquez-Medina JP, Keith AD, Khudyakov JI. 2021 Ontogeny of carbon monoxide-related gene expression in a deep-diving marine mammal. Front. Physiol. **12**, 762102. (10.3389/fphys.2021.762102) 34744798 PMC8567018

[52] Carter MID, Russell DJF, Embling CB, Blight CJ, Thompson D, Hosegood PJ, Bennett KA. 2017 Intrinsic and extrinsic factors drive ontogeny of early-life at-sea behaviour in a marine top predator. Sci. Rep. **7**, 15505. (10.1038/s41598-017-15859-8) 29138511 PMC5686064

[53] Crocker DE, Le Boeuf BJ, Costa DP. 1997 Drift diving in female northern elephant seals: implications for food processing. Can. J. Zool. **75**, 27-39. (10.1139/z97-004)

[54] Robinson PW et al. 2012 Foraging behavior and success of a mesopelagic predator in the northeast Pacific Ocean: insights from a data-rich species, the northern elephant seal. PLOS ONE **7**, e36728. (10.1371/journal.pone.0036728) 22615801 PMC3352920

[55] Jonsen ID, Grecian WJ, Phillips L, Carroll G, McMahon C, Harcourt RG, Hindell MA, Patterson TA. 2023 aniMotum, an R package for animal movement data: rapid quality control, behavioural estimation and simulation. Methods Ecol. Evol. **3**, 806-816. (10.1111/2041-210X.14060)

[56] National Oceanic and Atmospheric Administration. 2020 NOAA solar calculator: sunrise, sunset, and noon for any place on Earth, 2022. See https://gml.noaa.gov/grad/solcalc/.

[57] Robinson PW. 2009 Exploiting pelagic habitat: navigation, migration, and foraging success in the northern elephant seal (*Mirounga angustirostris*). Santa Cruz, CA: University of California.

[58] Robinson PW, Simmons SE, Crocker DE, Costa DP. 2010 Measurements of foraging success in a highly pelagic marine predator, the northern elephant seal. J. Anim. Ecol. **79**, 1146-1156. (10.1111/j.1365-2656.2010.01735.x) 20673236

[59] Wood SN. 2011 Fast stable restricted maximum likelihood and marginal likelihood estimation of semiparametric generalized linear models. J. R. Stat. Soc. Ser. B Stat. Methodol. **73**, 3-36.

[60] Pedersen EJ, Miller DL, Simpson GL, Ross N. 2019 Hierarchical generalized additive models in ecology: an introduction with mgcv. PeerJ **7**, e6876. (10.7717/peerj.6876) 31179172 PMC6542350

[61] R Core Team. 2022 R: a language and environment for statistical computing. Vienna, Austria: R Foundation for Statistical Computing.

[62] Irvine LG, Hindell MA, van den Hoff J, Burton HR. 2000 The influence of body size on dive duration of underyearling southern elephant seals (*Mirounga leonina*). J. Zool. **251**, 463-471. (10.1111/j.1469-7998.2000.tb00802.x)

[63] Scott R, Marsh R, Hays GC. 2014 Ontogeny of long distance migration. Ecology **95**, 2840-2850. (10.1890/13-2164.1)

[64] Noren SR, Jay CV, Burns JM, Fischbach AS. 2015 Rapid maturation of the muscle biochemistry that supports diving in Pacific walruses (*Odobenus rosmarus divergens*). J. Exp. Biol. **218**, 3319-3329. (10.1242/jeb.125757) 26347559

[65] Shero MR, Reiser PJ, Simonitis L, Burns JM. 2019 Links between muscle phenotype and life history: differentiation of myosin heavy chain composition and muscle biochemistry in precocial and altricial pinniped pups. J. Comp. Physiol. B **189**, 717-734. (10.1007/s00360-019-01240-w) 31616978

[66] Bennett KA, McConnell BJ, Moss SEW, Speakman JR, Pomeroy PP, Fedak MA. 2010 Effects of age and body mass on development of diving capabilities of gray seal pups: costs and benefits of the postweaning fast. Physiol. Biochem. Zool. PBZ **83**, 911-923. (10.1086/656925) 20969447

[67] Bryden MM, Lim GHK. 1969 Blood parameters of the southern elephant seal (*Mirounga leonina*, Linn.) in relation to diving. Comp. Biochem. Physiol. **28**, 139-148. (10.1016/0010-406X(69)91328-0) 5777362

[68] Thorson PH, Le Boeuf BJ. 1994 Developmental aspects of diving in northern elephant seal pups. In Elephant seals: population ecology, behavior, and physiology (eds BJ Le Boeuf, RM Laws), pp. 271-289. Berkeley, CA: University of California Press. (10.1525/9780520328150-017)

[69] Wilkinson IS, Bester MN. 1990 Duration of post-weaning fast and local dispersion in the southern elephant seal, *Mirounga leonina*, at Marion Island. J. Zool. **222**, 591-600. (10.1111/j.1469-7998.1990.tb06016.x)

[70] Richmond JP, Burns JM, Rea LD. 2006 Ontogeny of total body oxygen stores and aerobic dive potential in Steller sea lions (*Eumetopias jubatus*). J. Comp. Physiol. B **176**, 535-545. (10.1007/s00360-006-0076-9) 16514541

[71] Jouma'a J, Le Bras Y, Richard G, Vacquié-Garcia J, Picard B, El Ksabi N, Guinet C. 2016 Adjustment of diving behaviour with prey encounters and body condition in a deep diving predator: the southern elephant seal. Funct. Ecol. **30**, 636-648. (10.1111/1365-2435.12514)

[72] Schreer JF, Kovacs KM, O'Hara Hines RJ. 2001 Comparative diving patterns of pinnipeds and seabirds. Ecol. Monogr. **71**, 137-162. (10.1890/0012-9615(2001)071[0137:CDPOPA]2.0.CO;2)

[73] Halsey LG, Butler PJ, Blackburn TM. 2006 A phylogenetic analysis of the allometry of diving. Am. Nat. **167**, 276-287. (10.1086/499439) 16670986

[74] McIntyre T, de Bruyn PJN, Ansorge IJ, Bester MN, Bornemann H, Plötz J, Tosh CA. 2010 A lifetime at depth: vertical distribution of southern elephant seals in the water column. Polar Biol. **33**, 1037-1048. (10.1007/s00300-010-0782-3)

[75] Yano K, Stevens JD, Compagno LJV. 2004 A review of the systematics of the sleeper shark genus *Somniosus* with redescriptions of *Somniosus* (*Somniosus*) *antarcticus* and *Somniosus* (*Rhinoscymnus*) *longus* (Squaliformes: Somniosidae). Ichthyol. Res. **51**, 360-373. (10.1007/s10228-004-0244-4)

[76] Dragon A-C, Monestiez P, Bar-Hen A, Guinet C. 2010 Linking foraging behaviour to physical oceanographic structures: southern elephant seals and mesoscale eddies east of Kerguelen Islands. Prog. Oceanogr. **87**, 61-71. (10.1016/j.pocean.2010.09.025)

[77] Keates TR, Hazen EL, Holser RR, Fiechter J, Bograd SJ, Robinson PW, Gallo-Reynoso JP, Costa DP. 2022 Foraging behavior of a mesopelagic predator, the northern elephant seal, in northeastern Pacific eddies. Deep Sea Res. Part Oceanogr. Res. Pap. **189**, 103866. (10.1016/j.dsr.2022.103866)

[78] Klimley AP, Le Boeuf BJ, Cantara KM, Richert JE, Davis SF, Van Sommeran S, Kelly JT. 2001 The hunting strategy of white sharks (*Carcharodon carcharias*) near a seal colony. Mar. Biol. **138**, 617-636. (10.1007/s002270000489)

[79] Dahlheim ME, White PA. 2010 Ecological aspects of transient killer whales *Orcinus orca* as predators in southeastern Alaska. Wildl. Biol. **16**, 308-322. (10.2981/09-075)

[80] Condy PR, Aarde RJV, Bester MN. 1978 The seasonal occurrence and behaviour of killer whales *Orcinus orca*, at Marion Island. J. Zool. **184**, 449-464. (10.1111/j.1469-7998.1978.tb03301.x)

[81] Guinet C. 1991 Growth from birth to weaning in the southern elephant seal (*Mirounga leonina*). J. Mammal. **72**, 617-620. (10.2307/1382147)

[82] Keith M, Bester MN, Bartlett PA, Baker D. 2001 Killer whales (*Orcinus orca*) at Marion Island, Southern Ocean. Afr. Zool. **36**, 163-175. (10.1080/15627020.2001.11657134)

[83] Van Den Hoff J, Morrice MG. 2008 Sleeper shark (*Somniosus antarcticus*) and other bite wounds observed on southern elephant seals (*Mirounga leonina*) at Macquarie Island. Mar. Mammal Sci. **24**, 239-247. (10.1111/j.1748-7692.2007.00181.x)

[84] Klimley AP, Anderson SD, Pyle P, Henderson RP. 1992 Spatiotemporal patterns of white shark (*Carcharodon carcharias*) predation at the South Farallon Islands, California. Copeia **1992**, 680-690. (10.2307/1446143)

[85] Guinet C, Vacquié-Garcia J, Picard B, Bessigneul G, Lebras Y, Dragon AC, Viviant M, Arnould JPY, Bailleul F. 2014 Southern elephant seal foraging success in relation to temperature and light conditions: insight into prey distribution. Mar. Ecol. Prog. Ser. **499**, 285-301. (10.3354/meps10660)

[86] Slip DJ. 1995 The diet of southern elephant seals (*Mirounga leonina*) from Heard Island. Can. J. Zool. **73**, 1519-1528. (10.1139/z95-180)

[87] Martin C, Bentaleb I, Steelandt S, Guinet C. 2011 Stable carbon and nitrogen isotope variations in canine dentine growth layers of Kerguelen southern elephant seals. Mar. Ecol. Prog. Ser. **439**, 295-305. (10.3354/meps09331)

[88] Chaigne A, Authier M, Richard P, Cherel Y, Guinet C. 2013 Shift in foraging grounds and diet broadening during ontogeny in southern elephant seals from Kerguelen Islands. Mar. Biol. **160**, 977-986. (10.1007/s00227-012-2149-5)

[89] Walters A, Lea M-A, van den Hoff J, Field IC, Virtue P, Sokolov S, Pinkerton MH, Hindell MA. 2014 Spatially explicit estimates of prey consumption reveal a new krill predator in the Southern Ocean. PLoS ONE **9**, e86452. (10.1371/journal.pone.0086452) 24516515 PMC3905967

[90] Lübcker N, Reisinger RR, Oosthuizen WC, de Bruyn PJN, van Tonder A, Pistorius PA, Bester MN. 2017 Low trophic level diet of juvenile southern elephant seals *Mirounga leonina* from Marion Island: a stable isotope investigation using vibrissal regrowths. Mar. Ecol. Prog. Ser. **577**, 237-250. (10.3354/meps12240)

[91] Goetsch C, Conners MG, Budge SM, Mitani Y, Walker WA, Bromaghin JF, Simmons SE, Reichmuth C, Costa DP. 2018 Energy-rich mesopelagic fishes revealed as a critical prey resource for a deep-diving predator using quantitative fatty acid signature analysis. Front. Mar. Sci. **5**, 430. (10.3389/fmars.2018.00430)

[92] Yoshino K, Takahashi A, Adachi T, Costa DP, Robinson PW, Peterson SH, Hückstädt LA, Holser RR, Naito Y. 2020 Acceleration-triggered animal-borne videos show a dominance of fish in the diet of female northern elephant seals. J. Exp. Biol. **223**, jeb212936. (10.1242/jeb.212936) 32041802

[93] Miller PJO, Biuw M, Watanabe YY, Thompson D, Fedak MA. 2012 Sink fast and swim harder! Round-trip cost-of-transport for buoyant divers. J. Exp. Biol. **215**, 3622-3630. (10.1242/jeb.070128) 23014571

[94] Adachi T, Maresh JL, Robinson PW, Peterson SH, Costa DP, Naito Y, Watanabe YY, Takahashi A. 2014 The foraging benefits of being fat in a highly migratory marine mammal. Proc. R. Soc. B **281**, 20142120. (10.1098/rspb.2014.2120) PMC4241001 25377461

[95] Richard G, Vacquié-Garcia J, Jouma'a J, Picard B, Génin A, Arnould JPY, Bailleul F, Guinet C. 2014 Variation in body condition during the post-moult foraging trip of southern elephant seals and its consequences on diving behaviour. J. Exp. Biol. **217**, 2609-2619. (10.1242/jeb.088542) 24803471

[96] Crocker DE, Gales NJ, Costa DP. 2001 Swimming speed and foraging strategies of New Zealand sea lions (*Phocarctos hookeri*). J. Zool. **254**, 267-277. (10.1017/S0952836901000784)

[97] Arnbom T, Fedak MA, Boyd IL, McConnell BJ. 1993 Variation in weaning mass of pups in relation to maternal mass, postweaning fast duration, and weaned pup behaviour in southern elephant seals (*Mirounga leonina*) at South Georgia. Can. J. Zool. **71**, 1772-1781. (10.1139/z93-252)

[98] Hindell MA, McConnell BJ, Fedak MA, Slip DJ, Burton HR, Reijnders PJ, McMahon CR. 1999 Environmental and physiological determinants of successful foraging by naive southern elephant seal pups during their first trip to sea. Can. J. Zool. **77**, 1807-1821. (10.1139/z99-154)

[99] Kuhn CE, Crocker DE, Tremblay Y, Costa DP. 2009 Time to eat: measurements of feeding behaviour in a large marine predator, the northern elephant seal *Mirounga angustirostris*. J. Anim. Ecol. **78**, 513-523. (10.1111/j.1365-2656.2008.01509.x) 19040681

[100] Condit R, Reiter J, Morris PA, Berger R, Allen SG, Le Boeuf BJ. 2014 Lifetime survival rates and senescence in northern elephant seals. Mar. Mammal Sci. **30**, 122-138. (10.1111/mms.12025)

[101] McMahon CR, Burton HR. 2005 Climate change and seal survival: evidence for environmentally mediated changes in elephant seal, *Mirounga leonina*, pup survival. Proc. R. Soc. B Biol. Sci. **272**, 923-928. (10.1098/rspb.2004.3038) PMC1564088 16024347

[102] Jouma'a J, Orgeret F, Picard B, Robinson PW, Weimerskirch H, Guinet C, Costa DP, Beltran RS. 2023 Data from: Contrasting offspring dependence periods and diving development rates in two closely related marine mammal species. Dryad Digital Repository. (10.5061/dryad.tht76hf3t) PMC10762441 38179081

[103] SESjo. 2023 SESjo/ontodive: v0.1.4. Zenodo. (10.5281/zenodo.10368287)

[104] Jouma'a J, Orgeret F, Picard B, Robinson PW, Weimerskirch H, Guinet C, Costa DP, Beltran RS. 2024 Contrasting offspring dependence periods and diving development rates in two closely related marine mammal species. Figshare. (10.6084/m9.figshare.c.6971891) PMC10762441 38179081

